# Non-Coding RNAs as New Therapeutic Targets in the Context of Renal Fibrosis

**DOI:** 10.3390/ijms20081977

**Published:** 2019-04-23

**Authors:** Cynthia Van der Hauwaert, François Glowacki, Nicolas Pottier, Christelle Cauffiez

**Affiliations:** 1 EA 4483-IMPECS-IMPact of Environmental ChemicalS on Human Health, Univ. Lille, 59045 Lille CEDEX, France; cynthia.vanderhauwaert@gmail.com (C.V.d.H.); francois.glowacki@chru-lille.fr (F.G.); nicolas.pottier@univ-lille.fr (N.P.); 2 Département de la Recherche en Santé, CHU Lille, 59037 Lille, France; 3 Service de Néphrologie, CHU Lille, 59037 Lille, France; 4 Service de Toxicologie et Génopathies, CHU Lille, 59037 Lille, France

**Keywords:** non-coding RNAs, microRNAs, long non-coding RNAs, renal fibrosis, biomarkers, therapeutics targets

## Abstract

Fibrosis, or tissue scarring, is defined as the excessive, persistent and destructive accumulation of extracellular matrix components in response to chronic tissue injury. Renal fibrosis represents the final stage of most chronic kidney diseases and contributes to the progressive and irreversible decline in kidney function. Limited therapeutic options are available and the molecular mechanisms governing the renal fibrosis process are complex and remain poorly understood. Recently, the role of non-coding RNAs, and in particular microRNAs (miRNAs), has been described in kidney fibrosis. Seminal studies have highlighted their potential importance as new therapeutic targets and innovative diagnostic and/or prognostic biomarkers. This review will summarize recent scientific advances and will discuss potential clinical applications as well as future research directions.

## 1. Introduction

Chronic kidney disease (CKD) is increasingly recognized as a major public health concern. CKD prevalence has been estimated to be 8–16% worldwide [1]. In particular, CKD has been evaluated to affect more than 10% of the western population [2]. The common feature of CKD is renal fibrosis, which contributes to the progressive and irreversible decline in renal function and is associated with high morbidity and mortality.

Renal fibrosis, defined as an aberrant wound healing process in response to chronic injury, is characterized by the progressive and persistent accumulation of extracellular matrix components (ECM) in the kidney, ultimately leading to renal failure. As tissue scarring affects all compartments of the kidney, renal fibrosis is typically associated with glomerulosclerosis, arteriosclerosis and tubulointerstitial fibrosis [2]. Disruption of the epithelium and/or endothelium integrity during injury results in the activation of a complex cascade of molecular and cellular events. First, an inflammatory response initiates the release of profibrotic cytokines, chemokines and growth factors, which in turn promotes the proliferative phase of the scarring process characterized in particular by the recruitment and activation of fibroblasts into ECM-secreting myofibroblasts [3,4]. Finally, ECM accumulation results in the formation of a permanent fibrotic scar associated with renal tissue remodeling [5]. Once deposited, ECM components are further cross-linked and acquire resistance properties to degradation, precluding fibrosis resolution [6].

Although histological analysis of renal biopsies represents the gold standard to evaluate fibrosis, indirect biological parameters such as evolution of estimated Glomerular Filtration Rate are widely used in clinical practice for monitoring the progression of fibrotic lesions [7,8]. Furthermore, no specific treatment directly targeting fibrosis is currently approved [2]. Therefore, identifying new therapeutic targets and innovative diagnostic and/or prognostic biomarkers remains critical.

Recently, among the various mechanisms triggering fibrogenesis, non-coding RNAs (ncRNAs) have emerged as important regulators of this deleterious process [9,10,11,12,13].

In this review, we summarize the implication of ncRNAs in renal fibrosis and their potential value as either biomarkers or therapeutic targets, with an emphasis on microRNAs (miRNAs) and long non-coding RNAs (lncRNAs).

## 2. Non-Coding RNAs

**Figure 1:**
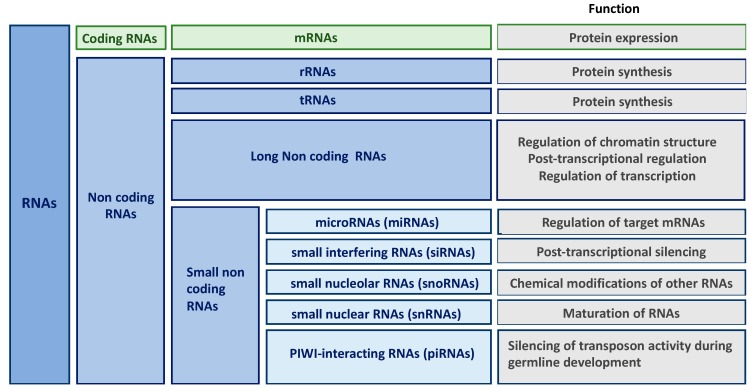
Classification and function of non-coding RNAs (ncRNAs).

New high-throughput technologies have revolutionized our understanding of the genome. Indeed, transcriptome of higher eukaryotic organism is far more complex than anticipated and contains large amounts of RNA molecules without coding potential (only 2% mRNAs in humans). Besides transfer and ribosomal RNAs that have been known since the 1950s, non-coding RNAs (ncRNAs) form a large and heterogenous class of RNA species involved in the regulation of gene expression. Non-coding RNAs are classified according to their length, localization and/or function into long non-coding RNAs (lncRNAs), microRNAs (miRNAs), small interfering RNAs (siRNAs), small nucleolar RNAs (snoRNAs), small nuclear RNAs (snRNAs) and PIWI-interacting RNAs (piRNAs) (Figure 1) [14,15,16,17]. Given that the role of some classes of ncRNAs (including siRNAs, snoRNAs or piRNAs) in kidney fibrosis remains largely unknown, this review will be restricted to miRNAs and lncRNAs.

### 2.1. microRNAs (miRNAs)

miRNAs are ncRNAs of about 22 nucleotides usually conserved between species and involved in post-transcriptional regulation of gene expression. Currently, about 2700 mature miRNAs have been identified in humans, regulating at least 60% of mRNAs (miRbase v.22.1, October 2018 [18]). As miRNAs are involved in a vast array of physiological processes, such as embryogenesis, cellular homeostasis and differentiation [19]. Their aberrant expression plays a causative role in most complex disorders such as cancer, cardio-vascular diseases and fibro-proliferative disorders [20,21,22,23].

About 60% of miRNAs are localized in intergenic regions and possess their own transcriptional unit [24]. Other miRNAs are localized in intron of coding genes and are either co-transcribed with their host genes or under the control of a specific promoter [25,26]. miRNAs are usually transcribed by RNA polymerase II into a primary transcript, termed pri-miRNA. This pri-miRNA is then processed into a pre-miRNA of about 70 nucleotides by a multiproteic complex, called microprocessor and composed of two subunits: The RNAse III endonuclease DROSHA and the RNA binding protein DGCR8 (DiGiorge Critical Region 8). The pre-miRNA is recognized by EXP5 (Exportin 5)-Ran-GTP and exported to the cytoplasm. The last step of maturation is catalyzed by the RNAse III DICER associated with TRBP (TAR RNA binding protein). The PAZ domain (PIWI-AGOZWILLE) of the complex allows the recognition and positioning of DICER, then the RNAse III domain cleaves the pre-miRNA loop, generating a 22-nucleotide miRNA duplex [27]. The association with an Argonaute protein into the RISC (RNA-induced silencing complex) allows the dissociation of the duplex [28]. The passenger strand (termed miRNA*) is then cleaved and released into the cytoplasm for degradation [29] whereas the guide strand, or mature miRNA, persists within RISC [30]. When both strands lead to a mature miRNA, they are identified by the suffix -3p or -5p depending on whether they come from the 3′or 5′ end of their precursor.

By preferentially binding on specific sequences, called “seed” sequences, which are mainly localized in the mRNA 3’-UTR (UnTranslated Region), mature miRNAs induce the degradation of the target mRNAs if miRNA-mRNA complementarity is perfect. However, this mechanism is minor in animals. Indeed, in the majority of cases, miRNAs regulate the expression levels of their target mRNAs by the recruitment of protein partners responsible for the activation of de-adenylation and de-capping associated with the 5′-to-3′ decay of mRNAs and possibly to translational repression mechanisms [30].

### 2.2. Long Non-Coding RNAs (lncRNAs)

In the human genome, about 30,000 lncRNA transcripts have been identified to date (GENCODE v29, [31]). LncRNAs, which are defined by being larger than 200 nucleotides, share common features with mRNAs, including being transcribed by RNA polymerase II, capped, cleaved, spliced, and polyadenylated [32,33].

**Figure 2:**
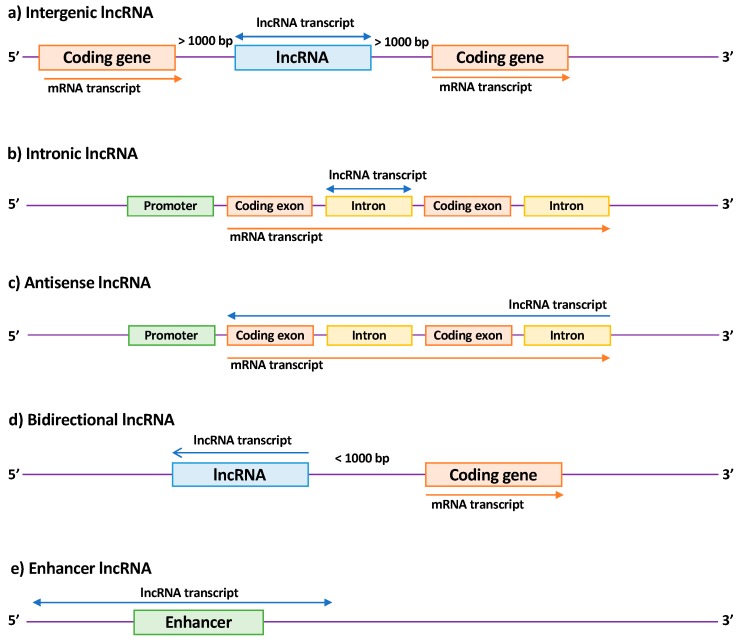
Classification of long non-coding RNAs (lncRNAs) according to their genomic location. (**a**) Intergenic lncRNAs are located between two coding genes; (**b**) intronic lncRNAs are transcribed entirely from introns of protein-coding genes; (**c**) antisense lncRNAs are transcribed from the antisense strand of a coding gene and overlap at least one exon; (**d**) bidirectional lncRNAs are localized within 1 kb of the promoter of a coding gene and oriented in the other direction; (**e**) enhancer lncRNAs are located in enhancer regions associated with a coding gene. Arrows indicate the direction of transcription.

LncRNA members are a heterogeneous family that can be subdivided according to their biogenesis loci into intergenic lncRNAs (lincRNAs), intronic lncRNAs, antisense lncRNAs (aslncRNA or natural antisense transcripts, NATs), bidirectional lncRNAs, and enhancer RNAs (eRNAs) [34,35,36,37] (Figure 2). Their functions are still poorly explored due to their subcellular localization [34] and their tissue- and temporal-specific expression [38]. Moreover, the low conservation of lncRNAs between species is a major obstacle to their identification and characterization in animal models [39]. Nevertheless, lncRNAs have been shown to display wide-ranging functions, probably due to their ability to bind to either DNA, RNA or protein. In particular, seminal functional studies have demonstrated their important role in the modulation of gene expression or DNA remodeling in physiological and pathological processes [32].

## 3. miRNAs Implicated in Renal Fibrosis

Among the various classes of ncRNAs, miRNAs have first retained the attention of the scientific community. Many studies that focused on miRNAs in renal fibrosis have been published and allowed the identification of about thirty miRNAs with either an anti-fibrotic or pro-fibrotic effect, also called “fibromiRs” [4,40]. While Table 1 outlines publications highlighting the major role of miRNAs in renal fibrosis, we will describe more precisely the role of few particularly well-characterized miRNAs.

### 3.1. miR-21

A large number of studies have emphasized the role of miR-21 in tissue fibrosis, notably in pulmonary [110], cardiac [111] or renal fibrosis [42]. The miR-21 gene locus is located within the *TMEM49* gene coding for Vacuole Membrane Protein 1 [112]. Interestingly, while miR-21 is one of the most highly expressed miRNAs in the healthy kidney [11], studies suggest that loss of miR-21 has no effect on development or healthy tissue function. This could be explained by its sequestration into an intracellular compartment. Nevertheless, in various stress conditions, miR-21 could be released into the cytoplasm to exert its regulatory functions [42,113]. In different experimental models such as the renal fibrosis (unilateral ureteral obstruction (UUO) mouse model, acute kidney injury (ischemia-reperfusion model) or diabetic nephropathy (*db*/*db* mice, streptozotocine-induced diabetes)), miR-21 is highly expressed in injured kidney regions [41,42,53,57]. Overexpression of miR-21 was also confirmed in renal allograft biopsies, in renal tissues of patients with IgA nephropathy or with Alport Syndrome exhibiting severe fibrotic injuries, particularly in regions enriched in fibroblasts/myofibroblasts, in the tubular epithelium and glomeruli [58,59,60]. The deleterious role of miR-21 in renal fibrosis was further explored using miR-21 null mice. Following UUO or ischemia-reperfusion injuries, miR-21^-/-^ mice exhibited less fibrosis. Moreover, authors showed that miR-21 is also involved in lipid metabolism and mitochondrial redox regulation [42]. While only a limited number of miR-21 target genes have been experimentally validated, miR-21 has been demonstrated to be involved in the regulation of critical signaling pathways related to fibrogenesis such as cellular proliferation (PTEN) [61], apoptosis (PDCD4, Bcl2) [43,62,63], regulation of cellular metabolism (PPARα, PHD2) [44,45,46], inflammation (MKK3) [47,48], ECM components (Reck, TIMP3) [49,50,51,52], TGF-β signaling pathway (Smad7) [54], angiogenesis (Reck, THSP-1, PHD2) [46,49,50,55] and autophagy (Rab11a) [56].

### 3.2. miR-214

miR-214 has been shown to act as a fibromiR in several types of tissue fibrosis, including liver [114] and heart fibrosis [115]. miR-214 has been also consistently associated with renal fibrosis. It is in particular upregulated by the activation of the transcription factor TWIST in response to hypoxia in renal tubular epithelial cells [71]. Moreover, Denby et al., using miR-214 null mice and the UUO model of kidney fibrosis, showed that miR-214 promotes renal fibrosis independently of TGF-β pathway [116]. Similarly, treatment with an antagonist of miR-214 before UUO protected against fibrogenesis without blocking Smad2/Smad3 activation and TGF-β signaling [116]. Other studies have mechanistically linked miR-214 pro-fibrotic function with the targeting of DKK3 (Wnt/β -catenin pathway) [72], CDH1 (EMT) [71] or PTEN (proliferation) [70].

### 3.3. miR-200 Family

Members of the miR-200 family include five members organized into two clusters, miR-200b/a/429 and miR-200c/141 [117,118]. In animal models of renal fibrosis either induced by UUO or gavage with adenine, miR-200 family members are consistently downregulated [98,99]. Indeed, the anti-fibrotic role of these miRNAs is mainly associated with epithelial differentiation [100] by protecting renal tubular cells from EMT process through the direct regulation of ZEB1/2 (zinc finger E-box-Binding homeobox proteins 1/2) and Ets-1 transcription factors [81,101,102,103]. Of note, miR-200 family is also involved in TGF-β signaling pathway by modulating TGF-β2 [98].

### 3.4. miR-29 Family

miR-29 family is composed of three members: miR-29a, miR-29b and miR-29c [119]. Expression of miR-29abc is invariably downregulated during fibrosis and their low expression is associated with the up-regulation of ECM-related genes [80]. In fact, decreased expression of miR-29 family members is a general downstream molecular event of TGF-β signaling, which is essential for the release of ECM components by fibroblasts, as miR-29 family members directly target multiple collagen isoforms and other ECM components [81,82,83].

In various animal models of renal fibrosis, expression of miR-29 members is downregulated regardless of the cause of injury [84,85]. Interestingly, TGF-β inhibited miR-29 expression not only in renal fibroblasts, but also in mesangial cells, epithelial cells and podocytes [85], suggesting that miR-29abc exert also an anti-fibrotic function in non-fibroblastic renal cells. For example, both Adam12 and Adam19 represent two pro-fibrotic targets of miR-29abc in renal tubular epithelial cells [84]. Similarly, Hu et al. showed that miR-29 targets, in renal tubular epithelial cells, PIK3R2, an effector of PI3K/AKT signaling pathway involved in EMT induced by Angiotensin II [86]. Nevertheless, the precise contribution of miR-29abc during fibrosis in non-stromal cells remains to be clarified, especially as Long et al. reported an increased expression of miR-29c in both podocytes and endothelial cells in a mouse model of diabetic nephropathy [87].

### 3.5. miR-192

Data regarding the role of miR-192 in renal fibrosis are currently controversial. In fact, miR-192 is upregulated in various mouse models of CKD such as diabetic nephropathy, UUO and IgA nephropathy [105,106,107,108]. In line with this, treatment with an antagonist of miR-192 protected against fibrosis through induction of Zeb1/2 in diabetic nephropathy mouse model [105]. By contrast, other studies have reported in vitro in renal cells exposed to TGF-β, in a mouse model of diabetic nephropathy as well as in renal tissue from patients exhibiting severe renal fibrotic lesions a downregulation of miR-192 [108,109]. Overall, data highlighting the versatile role of miR-192 in renal fibrosis represent a relevant example of the complexity of miRNA regulation mechanisms.

## 4. Long Non-Coding RNAs Implicated in Renal Fibrosis

Even if elucidation of the role of lncRNAs is still ongoing, it is now accepted that besides their involvement in physiological processes such as organ development, immunity or homeostasis, their modulation can occur in chronic multifactorial diseases [35].

In the context of fibrosis, few examples showing their pro-fibrotic role have been documented, such as MALAT1 in cardiac fibrosis, H19 and DNM3os in lung fibrosis, and MALAT1, lnc-LFAR1 and HIF1A-AS1 in liver fibrosis [120,121,122,123,124,125]. Although studies about lncRNAs and renal fibrosis are quite recent, their number has significantly increased in recent years. In particular, emerging data show that various lncRNAs are involved in renal fibrosis by playing a pro- or anti-fibrotic role (Table 2). Although many studies have shown a deregulation of lncRNA expression, we chose to only focus on mechanistic studies.

### 4.1. Errb4-IR

LncRNA Errb4-IR (np_5318), located in the ERBB4 intron region between the first and second exons, has been associated with renal fibrosis [134,135]. In the UUO mouse model, Errb4-IR was upregulated and strongly expressed in interstitial fibroblasts and injured tubular epithelial cells. Errb4-IR upregulation was also associated with fibrotic marker expression such as α-SMA or Collagen I. Moreover, in vivo silencing of Errb4-IR in the UUO mouse model significantly decreased fibrotic injuries [135]. Feng et al. also assessed the mechanisms underlying the fibrogenic role of Errb4-IR and showed that, in addition to being induced by TGF-β/Smad3 signaling, Errb4-IR directly targets Smad7, which exerts anti-fibrotic functions [135]. The pathological role of Errb4-IR in renal fibrosis was further confirmed in the context of diabetic nephropathy [136] by demonstrating that Errb4-IR also targets miR-29b, a well-established anti-fibrotic miRNA.

### 4.2. HOTAIR

HOTAIR (HOX transcript antisense intergenic RNA), embedded in the HOXC locus, is known to drive cancerogenesis [156]. Recently, two studies have demonstrated that HOTAIR is upregulated in renal fibrosis. In the UUO rat model, HOTAIR overexpression was associated with an upregulation of fibrotic and EMT markers as well as with a downregulation of miR-124, a miRNA involved in EMT and acting as a negative regulator of Nocth1 signaling pathway [130,131]. Moreover, lentiviral-mediated overexpression of HOTAIR in UUO rats, led to more severe injuries, such as inflammation, necrosis and collagen deposits, an elevated score of renal fibrosis and an overexpression of fibrotic markers compared to UUO alone. Mechanistically, it has been shown that HOTAIR activates the Notch1/Jagged1 signaling pathway by acting as a ceRNA (competing endogenous RNA—an lncRNA–miRNA duplex which prevents binding miRNA to its target and thus the target inhibition) with miR-124, which targets Notch1 and JAG1, and thereby promotes renal fibrosis [157,158].

### 4.3. Gm4419

In diabetic nephropathy, lincRNA Gm4419 was found to be involved in renal fibrosis. More precisely, in mesangial cells in high glucose conditions, overexpression of GM4419 was associated with fibrosis, inflammation and cell proliferation. Authors demonstrated that the NF-κB signaling pathway, which plays an important role in fibrogenesis and inflammation [140], was activated by GM4419 by interacting with its subunit p50. Moreover, p50 and GM4419 could have a synergistic effect in the inflammatory pathway [141].

## 5. New Therapeutic Targets and Innovative Biomarkers

### 5.1. New Therapeutic Targets

To date, the lack of specific anti-fibrotic therapies remains a critical need in clinical practice. As ncRNAs are involved in many critical pathogenic processes driving renal fibrosis, they represent attractive therapeutic targets. Currently, two strategies can be applied to manipulate ncRNA expression levels: The first relies on restoring the expression of a ncRNA when its level is decreased, the second is related to inhibiting the function of a ncRNA when its expression is increased.

#### 5.1.1. miRNAs as Therapeutic Targets

To restore miRNA function, miRNA mimics or pre-miRNA have been developed. A modified synthetic RNA is introduced into cells as a duplex consisting of one strand identical to the mature miRNA of interest (guide strand) and the second antisense strand with a lower stability [159]. In addition, chemical modifications such as 2′-Fluoro bases have been developed to increase the stability of the guide strand without interfering with the RISC complex [160]. Other modifications include the use of 5′-O-methyl bases on the second strand to limit its incorporation into RISC complex [161]. Finally, addition of cholesterol-like molecules improves the duplex cellular internalization [159]. Although the use of such tools is widely developed for in vitro models, their application in vivo is hampered by delivery [11]. Other approaches involved gene therapy techniques, using notably AAV-mediated miRNA delivery (adeno-associated virus). Indeed, AAVs allow the restoration of the physiological expression level of miRNA with low toxicity and without integration into the genome in a specific tissue or cellular type [159,162].

Such strategies have been successfully applied in preclinical mouse models of tissue fibrosis, including bleomycin-induced lung fibrosis, but still need to be evaluated in the context of kidney fibrosis [163]. The renal tissue is indeed accessible to AAV gene delivery by different routes, including injection through the renal artery, injection into the parenchyma and retrograde injection via the ureter.

Concerning miRNA inhibition, several strategies have been developed, especially antisense oligonucleotides (termed antimiRs) which are widely used in preclinical models of tissue fibrosis and have also entered clinical trials [164]. These molecules are also chemically modified in order to improve their affinity, pharmacokinetics, stability and cellular entrance. The major modifications include the addition on the ribose of particular groups such as 2′O-Methyl, 2′O-Methoxyethyl or 2′-Fluoro and also inclusion of bicyclic structures which lock the ribose into its preferred 3′ endo conformation and increase base-pairing affinity such as methylene bridging group, also known as LNA (locked nucleic acid). Such ribose modifications allow a reduction in the size of antimiRs without loss of affinity and specificity. Finally, backbone modifications such as phosphorothioate linkages or the addition of morpholino structures enhance nuclease resistance [165].

Finally, target site blockers (TSB) inhibit miRNA function by specifically preventing interaction between a miRNA and its target [166,167]. One advantage of this strategy relies on its specificity, as it does not affect expression of the other target genes, and thus reduces the risk of side effects.

In the context of renal fibrosis, proof-of-concept for miRNA targeting has been demonstrated for several fibromiRs. In particular, results indicated that miR-214 antagonism was associated with less fibrotic lesions in the UUO mouse model [116]. In addition, an miR-21 antagonism injection prevented fibrotic injuries in UUO [42], diabetic nephropathy [168] or Alport [169] mouse models. Moreover, Regulus Therapeutics has developed a phase II clinical trial with a miR-21 antagonist in patients with Alport syndrome (RG-012; Regulus Therapeutics Inc.; clinical trial: NCT02855268). This drug candidate has currently received the orphan drug status from the FDA and the European Commission for the treatment of this rare disease.

#### 5.1.2. lncRNAs as Therapeutic Targets

LncRNA deregulation is also viewed as an important driver of renal fibrosis, suggesting their potential value as therapeutic targets. Given their extensive secondary structures and their localization in nuclear and/or cytoplasmic compartments [15,34], pharmacological modulation of lncRNAs is more complex and, until recently, the options for targeting lncRNAs were limited. Moreover, the low conservation of lncRNAs between species is a major obstacle for preclinical validation [39,170]. However, recently, conceptual and technological advances in antisense oligonucleotide therapy offer new pharmacological options to modulate the expression or the function of lncRNAs. For example, the development of technologies including GapmeR-mediated lncRNA silencing, CRISPR inhibition or aptamers directed against lncRNA secondary structure represent novel opportunities to improve lncRNA knowledge and clinical translation [171].

In the context of renal fibrosis, lncRNA modulation remains an almost unexplored area. However, Kato et al. have used GapmeRs, an antisense oligonucleotide technology that induces target degradation in the nuclear compartment by recruiting RNAse H [172], in a mouse model of diabetic nephropathy. Interestingly, injection of such GapmeRs against lnc-MGG induced a decreased expression of profibrotic genes (TGF-β1, Col1a2, Col4a1, Ctgf) and prevented glomerular fibrosis, podocyte death and hypertrophy in diabetic mice [173]. Otherwise, few studies have investigated the opportunity to downregulate lncRNA expression using short hairpin RNAs (shRNAs) by delivery of plasmids or through viral or bacterial vectors in vivo [174]. Indeed, targeting of Errb4-IR was shown to improve renal fibrosis in the *db*/*db* mouse model [136]. Moreover, in a UUO mouse model, Arid2-IR was also successfully inhibited by a shRNA [144].

## 6. Biomarkers

Histological examination of biopsied tissue is considered the reference method for the diagnosis and staging of kidney fibrosis [8]. However, as percutaneous tissue sampling of either native kidney or allograft remains associated with patient discomfort, risk for complications, histopathological interpretation variability and high cost [175], the development of alternative non-invasive diagnostic or prognostic biomarkers is an important clinical issue [176]. Interestingly, ncRNAs that have been extensively reported to be dysregulated in fibrotic tissues, have also been detected in a large panel of human biological fluids including serum, plasma and urine [177,178,179].

### 6.1. miRNAs

In order to discover relevant biomarkers, miRNA profiling in several biofluids has been performed. Urine is a particularly interesting matrix to explore kidney function, even if miRNAs in urine are less abundant than in plasma or serum, since RNase activity has been reported to be quite high in urine [180]. Cardenas-Gonzalez et al. have screened more than 2000 urinary miRNAs from patients with CKD. In particular, this study demonstrated that downregulation of miR-2861, miR-1915-3p and miR-4532 was associated with a poorer renal function, interstitial fibrosis and tubular atrophy in diabetic nephropathy [181]. Another study profiled more than 1800 miRNAs in urine samples from patients with acute kidney injury. Among the 378 detected miRNAs, 19 were upregulated in patients with acute kidney injury, including miR-21, miR-200c and miR-423 [182]. Sonoda et al. showed that miR-9a, miR-141, miR-200a, miR-200c and miR-429 from exosomes in rat urine were upregulated following ischemia-reperfusion injury [183]. Moreover, Khurana et al. identified nine upregulated miRNAs (let-7c-5p, miR-222–3p, miR-27a-3p, miR-27b-3p, miR-296-5p, miR-31-5p, miR-3687, miR-6769b-5p and miR-877-3p) and seven downregulated miRNAs (miR-133a, miR-133b, miR-15a-5p, miR-181a-5p, miR-34a-5p, miR-181c-5p and miR1-2) in urine exosomes from patients with CKD compared to healthy controls [184]. Finally, other studies showed that dysregulation of urinary miR-29c, miR-21 and miR-200b was correlated with renal fibrotic injuries in patients with CKD or in renal transplanted patients [185,186,187]. Altogether, these data indicate that detection of miRNAs in the urine could reflect the degree of the renal aggression [188].

Finally, miRNAs were also detectable in serum and, more specifically, in renal transplanted patients serum level expression of miR-21 was found to be associated with the severity of renal fibrosis injuries [58,189,190]. While promising, the clinical use of circulating miRNAs as biomarkers remains tempered by quality control and normalization issues. For example, hemolysis needs to be perfectly avoided since miRNAs can be released from blood cells, thus affecting the amount of detected circulating miRNAs [191]. Furthermore, no standard endogenous control to normalize circulating miRNA levels has been clearly established and this concern is still debated [192,193]. The development of new technologies such as digital PCR (dPCR) are particularly interesting as this approach allows an absolute quantification without internal normalization [194,195].

### 6.2. lncRNAs

Although the expression of many lncRNAs has been evaluated in the context of fibrosis, their validation as biomarkers is at an earlier stage than miRNAs. Nevertheless, identifying novel lncRNAs as biomarkers is of great interest, since lncRNAs are highly stable in biofluids, especially when they are included in exosomes or in apoptotic bodies [179] and could be present in extracellular vesicles [196]. In renal fibrosis, Sun et al. compared the lncRNA profile in renal tissues and urines of UUO rats. Seven lncRNAs (five upregulated and two downregulated) were similarly modulated in renal tissues and urine. In addition, several conserved Smad3 binding motifs were identified in the sequence of the five upregulated lncRNAs [138]. Altogether, these results raise the possibility of using urinary lncRNAs as non-invasive biomarkers of renal fibrosis. Otherwise, Gao et al. found that in the serum of patients with diabetic nephropathy, the upregulation of lncRNA NR_033515 was correlated with NGAL and KIM1 serum levels, and the severity of the disease [145]. While both of these studies highlighted the potential of lncRNAs as non-invasive biomarkers for renal fibrosis, further studies are clearly required for the robust identification and validation of diagnostic and prognostic biomarkers.

## 7. Future Directions

**Figure 3:**
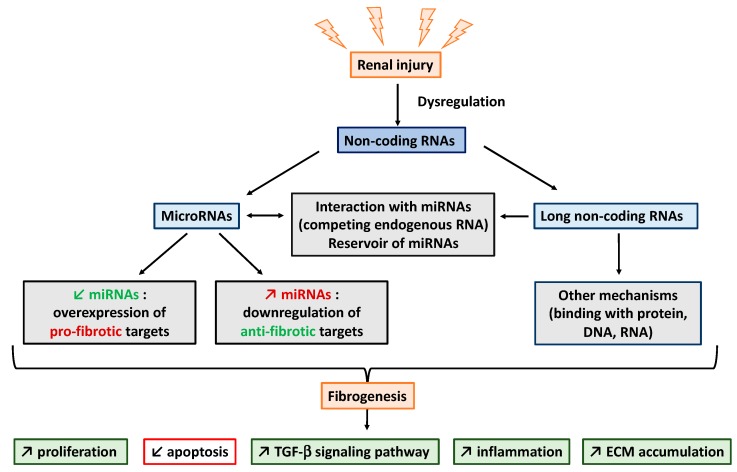
General mechanisms of non-coding RNAs involved in kidney fibrosis.

NcRNAs, including the well-known miRNAs and the emerging lncRNAs, have been described to be implicated in a large number of physiological and pathological processes (Figure 3). In particular, their modulation between normal and fibrotic renal tissues not only strongly suggests that ncRNAs are involved in the development and the progression of kidney fibrosis, but also that ncRNAs may represent promising biomarkers. However, in contrast to miRNAs, the underlying mechanisms of most of the identified lncRNAs are yet to be determined. Considering both technological advances and rising scientific enthusiasm in lncRNA biology, we foresee that major discoveries will soon be achieved regarding the role of lncRNAs in kidney fibrosis.

The proof of concept of ncRNA expression modulation to treat fibroproliferative disorders has been elegantly demonstrated. Clinical translation of these potential new therapeutic targets should be considered a research priority and will undoubtedly represent a gold mine of new therapeutic targets that may lead to the development of novel anti-fibrotics.

## Figures and Tables

**Table 1:** Summary of miRNAs involved in renal fibrosis.

Regulation	miRNA	Models	Gene Target	References
Up	miR-21	Renal tissues from kidney transplanted patients Renal tissues from patients with IgA nephropathy Renal tissues from patients with Alport Syndrome UUO mouse model DN mouse model Ichemia reperfusion mouse model RPTEC cells Mesangial cells	PTEN, SMAD7, PPARA, PDCD4, BCL2, PHD2, MKK3, RECK, TIMP3, THSP1, RAB11A	[41,42,43,44,45,46,47,48,49,50,51,52,53,54,55,56,57,58,59,60,61,62,63]
	miR-22	DN rat model RPTEC cells	PTEN	[64]
	miR-135a	Serum and renal tissues from patients with DN DN mouse model Mesangial cells	TRPC1	[65]
	miR-150	renal tissue from patients with lupus nephritis RPTEC cells Mesangial cells	SOCS1	[66]
	miR-155	UUO mouse model RPTEC cells	PDE3A	[67,68]
	miR-184	UUO mouse models RPTEC cells	HIF1AN	[69]
	miR-214	UUO mouse model DN mouse model RPTEC cells Mesangial cells	DKK3, CDH1, PTEN	[70,71,72]
	miR-215	DN mouse model Mesangial cells	CTNNBIP1	[73]
	miR-216a	DN mouse model Mesangial cells	YBX1	[74]
	miR-324	Rat model of nephropathy (Munich Wistar Fromter rats) RPTEC cells	PREP	[75]
	miR-433	UUO mouse model RPTEC cells	AZIN1	[76]
	miR-1207	RPTEC cells Mesangial cells	G6PD, PMEPAI1, PDK1, SMAD7	[77]
Down	let-7 family	DN mouse model RPTEC cells	HMGA2, TGFBR1	[78,79]
	miR-29 family	UUO mouse model Adenine gavage in mice Chronic renal failure rat model (5/6e nephrectomy) DN mouse model RPTEC cells Endothelial cells Podcytes HEK293 treated with ochratoxin A	COL, FN1, AGT, ADAM12, ADAM19, PIK3R2	[80,81,82,83,84,85,86,87,88]
	miR-30	Renal tissues from kidney transplanted patients UUO mouse model DN mouse model RPTEC cells	CTGF, KLF11, UCP2	[89,90,91]
	miR-34 family	UUO mouse model RPTEC cells	NOTCH1/JAG1	[92]
	miR-152	RPTEC cells	HPIP	[93]
	miR-181	UUO mouse model	EGR1	[94]
	miR-194	Ischemia reperfusion mouse model RPTEC cells	RHEB	[95]
	miR-200 family	UUO mouse model Adenine gavage in mice RPTEC cells	ZEB1/2, ETS1	[96,97,98,99,100,101,102]
	miR-455	DN rat model RPTEC cells Mesangial cells	ROCK2	[103]
Down/Up (controversial)	miR-192	UUO mouse model DN mouse model IgA nephropathy mouse model RPTEC cells	ZEB1/2	[104,105,106,107,108,109]

Abbreviations: UUO (ureteral unilateral obstruction); RPTEC (renal proximal tubular epithelial cells); DN (diabetic nephropathy).

**Table 2:** LncRNAs involved in kidney fibrosis.

Regulation	lncRNA	Models	Functions/Mechanisms	Consequences	References
Up	**LOC105375913**	**Renal tissue of patients with segmental glomeruloscleoris** **RPTEC cells**	**Binding to miR-27b and leading to Snail expression**	**Pro-fibrotic**	[126]
	**LINC00667**	**Renal tissue of patients with chronic renal failure** **Chronic renal failure rat model (partial nephrectomy)** **RPTEC cells**	**Binding to Ago2, targeting miR-19b-3p**	**Pro-fibrotic**	[127]
	NEAT1	DN rat model Mesangial cells		Pro-fibrotic and increase of proliferation	[128]
	**Lnc-TSI (AP000695.6** **or ENST00000429588.1)**	**RPTEC cells** **UUO mouse model** **Ischemia-reperfusion mouse model** **Renal tissue of patients with IgA nephropathy**	**Synergic binding to Smad3**	**Anti-fibrotic**	[129]
	**HOTAIR**	**UUO rat model** **RPTEC cells**	**Acting as a ceRNA with miR-124: activation of Jagged1/Nocth1 signaling**	**Pro-fibrotic**	[130,131]
	**LINC00963**	**Chronic renal failure rat model (5/6e nephrectomy)**	**Inhibition of FoxO signaling pathway by targeting FoxO3a**	**Pro-fibrotic**	[132]
	TCONS_00088786	UUO mouse model RPTEC cells	Possibly regulation of miR-132 expression	Pro-fibrotic	[133]
	**Errb4-IR** **(np-5318)**	**UUO mouse model** **Anti GBM mouse model** **RPTEC cells** **DN mouse model** **Mesangial cells**	**Downstream of TGFb/Smad3 pathway by binding Smad7 gene** **Binding to miR-29b**	**Pro-fibrotic**	[134,135,136]
	CHCHD4P4	Stone kidney mouse model RPTEC cells		Pro-fibrotic	[137]
	TCONS_00088786	UUO rat model RPTEC cells		Pro-fibrotic	[138]
	TCONS_01496394	UUO rat model RPTEC cells		Pro-fibrotic	[138]
	ASncmtRNA-2	DN mouse model Mesangial cells		Pro-fibrotic	[139]
	**LincRNA-Gm4419**	**DN mouse model** **Mesangial cells**	**Activation of NFkB/NLRP3 pathway by interacting with p50**	**Pro-fibrotic and pro-inflammatory**	[140,141]
	**H19**	**UUO mouse model** **RPTEC cells**	**Acting as a ceRNA with miR-17 and fibronectin mRNA**	**Pro-fibrotic**	[142]
	RP23.45G16.5	UUO mouse model RPTEC cells		Pro-fibrotic	[143]
	AI662270	UUO mouse model RPTEC cells		No significative effect	[143]
	**Arid2-IR** **(np-28496)**	**UUO mouse model** **RPTEC cells**	**Smad3 binding site in Arid2-IR promoter** **Promoting NF-** **κB signaling**	**Pro-fibrotic and pro-inflammatory effects**	[144]
	np-17856	UUO mouse model Glomerulonephritis mouse model	Smad3 binding site	Pro-fibrotic and pro-inflammatory	[134]
	**NR_033515**	**Serum of patients with diabetic nephropathy** **Mesangial cells**	**Targeting miR-743b-5p**	**Pro-fibrotic and promotes proliferation**	[145]
	**MALAT1**	**DN mouse model** **Podocytes**	**Binding to SRSF1** **Targeting by** **β-catenin**	**Pro-fibrotic**	[146]
	Gm5524	DN mouse model Podocytes		Autophagy increase and apoptosis decrease	[147]
	WISP1-AS1	RPTEC cells	Modulating ochratoxin-A-induced Egr-1 and E2F activities	Cell viability increase	[148]
Down	Gm15645	DN mouse model Podocytes		Autophagy decrease and apoptosis increase	[147]
	**CYP4B1-PS1-001** **(ENSMUST00000118753)**	**DN mouse model** **Mesangial cells**	**Enhancing ubiquitination and degradation of nucleolin**	**Anti-fibrotic and anti-proliferative**	[149,150]
	3110045C21Rik	UUO mouse model RPTEC cells		Anti-fibrotic	[143]
	ENSMUST00000147869	DN mouse model Mesangial cells	Associated with Cyp4a12a	Anti-fibrotic and anti-proliferative	[151]
	**lincRNA 1700020I24Rik** **(ENSMUSG00000085438)**	**DN mouse model** **Mesangial cells**	**Binding to miR-34a-5p. Inhibition of Sirt1/ HIF-1α signal pathway by targeting miR-34a-5p.**	**Anti-fibrotic**	[152]
	**MEG3**	**RPTEC cells**		**Anti-fibrotic**	[153]
	**ZEB1-AS1**	**DN mouse model** **RPTEC cells**	**Promoting Zeb1 expression by binding H3K4 Methyltransferase MLL1**	**Anti-fibrotic**	[154]
	**ENST00000453774.1**	**Renal tissue of patients with renal fibrosis** **UUO mouse model** **RPTEC cells**		**Anti-fibrotic**	[155]

Note: Studies in bold are mechanistic studies. Abbreviations: UUO (ureteral unilateral obstruction); RPTEC (renal proximal tubular epithelial cells); DN (diabetic nephropathy)

## Abbreviations

AAV	Adeno-associated-virus
ceRNA	Competing endogenous RNA
CKD	Chronic kidney disease
DN	Diabetic nephropathy
ECM	Extracellular matrix
FDA	Food and Drug Administration
HOTAIR	HOX transcript antisense intergenic RNA
lncRNA	Long non-coding RNA
miRNA	microRNA
ncRNA	non-coding RNA
RISC	RNA-induced silencing complex
RPTEC	Renal proximal tubular epithelial cell
shRNA	Short hairpin RNA
UUO	Ureteral unilateral obstruction
